# Singlet oxygen mediated DNA degradation by copper nanoparticles: potential towards cytotoxic effect on cancer cells

**DOI:** 10.1186/1477-3155-9-9

**Published:** 2011-03-25

**Authors:** Gregor P Jose, Subhankar Santra, Swadhin K Mandal, Tapas K Sengupta

**Affiliations:** 1 Department of Biological Sciences, Indian Institute of Science Education and Research-Kolkata, Mohanpur Campus, P.O. BCKV Main Office, Mohanpur - 741252, India; 2 Department of Chemical Sciences, Indian Institute of Science Education and Research-Kolkata, Mohanpur Campus, P.O. BCKV Main Office, Mohanpur - 741252, India

## Abstract

The DNA degradation potential and anti-cancer activities of copper nanoparticles of 4-5 nm size are reported. A dose dependent degradation of isolated DNA molecules by copper nanoparticles through generation of singlet oxygen was observed. Singlet oxygen scavengers such as sodium azide and Tris [hydroxyl methyl] amino methane were able to prevent the DNA degradation action of copper nanoparticles confirming the involvement of activated oxygen species in the degradation process. Additionally, it was observed that the copper nanoparticles are able to exert cytotoxic effect towards U937 and Hela cells of human histiocytic lymphoma and human cervical cancer origins, respectively by inducing apoptosis. The growth characteristics of U937 and Hela cells were studied applying various concentrations of the copper nanoparticles.

## Findings

Nanotechnology is one of the most rapidly growing disciplines with a wide range of applications, especially in electronics, information technology, sensor development, catalysis, and biomedical sciences [1-5]. Nanoparticles have a specific capacity for drug loading, efficient photoluminescence ability and are therefore important materials in the targeted delivery of imaging agents and anti-cancer drugs [6-9]. The extremely small size of the nanoparticles makes them to be utilized for potential target oriented delivery of nanomedicines in organs such as the brain, which are normally protected by specialized barriers (such as the blood-brain barrier). If these trends continue with nanomedicines, humans will be continuously benefited using exceedingly improved nanomaterials with diverse properties to act at the interface between nanotechnology and biology [10].

Continuous demand for new anti-cancer drugs has stimulated chemotherapeutic research based on the use of metals since potential drugs developed in this way may be less toxic and more prone to exhibit anti-proliferative activity against tumors [11,12]. Transition metal complexes have been extensively studied for their nuclease-like activity using the redox properties of the metal and dioxygen to produce reactive oxygen species to promote DNA cleavage by direct strand scission or base modification [13]. More recent trend in this area has been testing of metal nanoparticles such as gold and platinum nanoparticles for DNA degradation studies [14,15]. Use of metal nanoparticles can be in particular advantageous in generating singlet oxygen [16,17]. A recent report by Geddes and coworkers demonstrated that the presence of metal nanoparticles can enhance singlet oxygen generation [18]. The enhanced electromagnetic fields in proximity to metal nanoparticles are the basis for the increased absorption and various computational methods are available to predict the extent of absorption and the relative increase in singlet oxygen generation from photosensitizers [19,20]. Although a number of well-defined copper (II) complexes exhibited their DNA degradation capabilities [21,22], there are no reports on *in vitro *study of DNA degradation using copper nanoparticles (CuNPs). A very recent study by Midander and coworkers reported the effect of CuNPs inducing single stranded breaks in the cultured human lung cells [23]. Earlier studies showed potent cytotoxic, genotoxic and toxicological activities of CuNPs *in vivo *[23,24] and in cultured cancer cell lines [12]. However, a systematic study using CuNPs on DNA degradation and cytotoxicity towards different cancer cells are missing till to date to the best of our knowledge.

In this communication, a dose dependent DNA degradation action of copper nanoparticles (CuNPs) on isolated DNA molecules at 37°C is reported. Singlet oxygen scavengers such as sodium azide and Tris [hydroxyl methyl] amino methane were found to prevent the DNA degradation action of CuNPs and this observation confirms the involvement of activated oxygen species in the degradation process. Fluorescence quenching studies and densitometry analysis revealed the affinity of the interaction of DNA with CuNPs and the kinetics of DNA degradation by CuNPs, respectively. This study demonstrates that CuNPs can induce singlet oxygen mediated DNA damage and thus to be considered as potent cytotoxic agent to target cancer cells for the therapeutic applications. In fact, it was observed that the CuNPs could exert cytotoxic effect towards U937 and Hela cell lines of human lymphoma and cervical cancer origins, respectively by inducing apoptosis.

**Figure 1:**
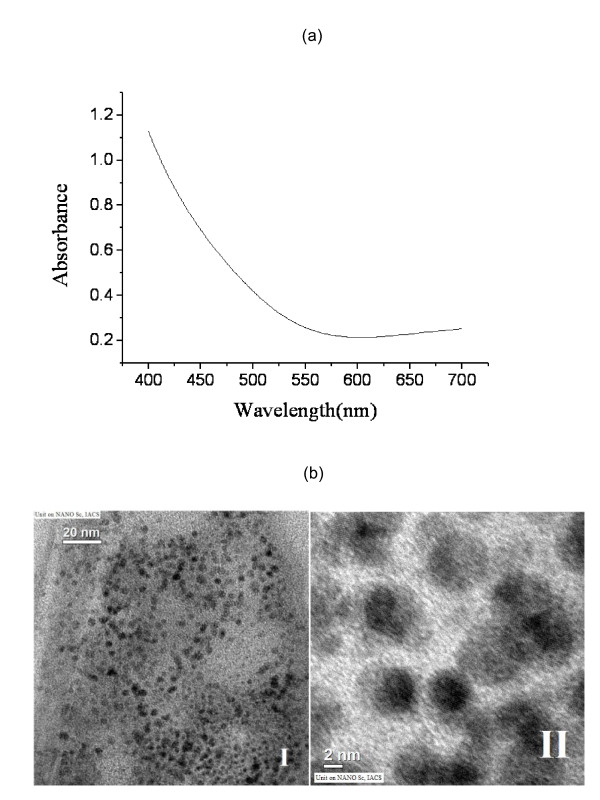
**Characterization of copper nanoparticles**. (a) UV-Vis spectrum of the CuNPs exhibiting a Mie scattering profile and (b) TEM images: left one (I) displays a TEM image of CuNPs and right one (II) displays a higher magnification image of the same revealing the presence of well dispersed particles having size between 4-5 nm.

**Figure 2:**
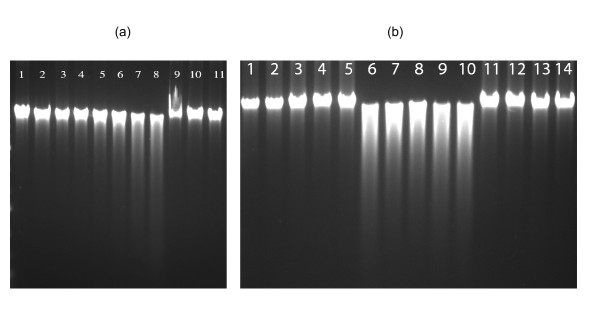
**Singlet oxygen mediated DNA degradation by copper nanoparticles**. (a) Dose dependent DNA degradation action of CuNPs. Lane 1 - Control DNA, Lane 2 - DNA + supernatant, Lanes 3 to 8 - DNA + 50, 100, 200, 300, 400, 500 μM CuNPs, respectively, Lane 9 - DNA + 500 μM CuSO_4_, Lane 10 - DNA + 4 mM sodium citrate, Lane 11 - DNA + 100 μM sodium borohydride and (b) Comparison of the ROS scavenging activity. Lane 1 - DNA alone, Lanes 2 to 5 - DNA + DMSO, D-mannitol, sodium azide, Tris (all 0.2 M) respectively, Lane 6 - DNA + 500 μM CuNPs, Lanes 7 to 14 - DNA + 500 μM CuNPs and DMSO 0.1 M, DMSO 0.2 M, D-mannitol 0.1 M, D-mannitol 0.2 M, azide 0.1 M, azide 0.2 M, Tris 0.1 M, Tris 0.2 M, respectively.

**Figure 3:**
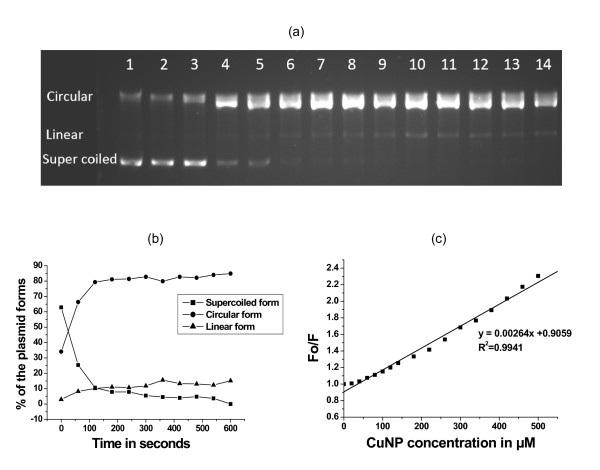
**DNA degradation kinetics and DNA binding by Copper nanoparticles**. (a) Time dependent plasmid degradation (Lane 1- DNA alone, Lane 2- DNA+ 0.2 M Tris, Lane 3-DNA+ 0.2 M Tris + 500 μM CuNPs, Lanes 4 to 14 - DNA + 500 μM CuNPs incubated for 10, 60, 120,180, 240, 300, 360, 420, 480, 540, 600 seconds followed by addition of 0.2 M Tris; (b) Time dependent change in the plasmid forms and (c) Binding of CuNPs with DNA shown by Stern-Volmer plot of DNA-EB in presence of different concentration of CuNPs.

The CuNPs were prepared in aqueous solution by reducing Cu^2+ ^ions with sodium borohydride in the presence of sodium citrate as a capping agent following a modified literature method [25]. Characterization of copper nanopaticles was carried out by UV-Vis spectroscopy and transmission electron microscopic (TEM) studies (Figure 1). The average nanoparticles size has been found to be 4-5 nm. The effect of copper nanoparticles on bacterial genomic DNA isolated from *Escherichia coli *was tested by treating the DNA with CuNPs of gradually increasing concentrations ranging from 50-500 μM for 100 minutes at 37°C in phosphate buffered saline (PBS) maintained at pH 7.4. After the incubation, the fate of DNA was analyzed by agarose gel electrophoresis. It was observed that the CuNPs induced DNA degradation and the degree of DNA degradation was directly proportional to the concentration of CuNPs (Figure 2a). Copper sulphate, sodium citrate, sodium borohydride solutions as well as the supernatant of CuNPs dispersion were also incubated with DNA as controls and none of these components were able to degrade DNA. This observation confirmed that CuNPs were solely responsible for DNA degradation (Figure 2a). Furthermore, the chemical scavengers which can scavenge active species in the reaction mixture were used to unravel the mechanistic pathway of degradation process. Scavengers included dimethyl sulphoxide and D-mannitol (hydroxyl free radical scavengers), sodium azide and Tris [hydroxyl methyl] amino methane (singlet oxygen scavengers) [26-28]. It was observed that sodium azide (0.1 M, 0.2 M) and tris [hydroxyl methyl] amino methane (0.1 M, 0.2 M) completely inhibited the CuNPs mediated DNA degradation (Figure 2b, Lanes 10-14). On the other hand, D-mannitol (0.1 M, 0.2 M), dimethyl sulphoxide (0.1 M, 0.2 M) were unable to prevent the DNA degradation completely (Figure 2b, Lanes 7-10). These results clearly indicate that CuNPs induced DNA degradation proceeds through a singlet oxygen mediated mechanism. In order to calculate the rate constant of the DNA degradation by copper nanoparticles, pET28b plasmid DNA was treated with 500 μM CuNPs for different time intervals and the different forms of plasmid DNA (supercoiled, circular and linear) were analyzed by agarose gel electrophoresis. The percentages of the different forms of plasmid DNA were estimated by densitometric analysis with the help of "Quantity one" software (Figure 3a and 3b). The supercoiled to circular form conversion curve was fitted in to the first order exponential decay equation [29]. The decay constant was found to be 0.0177 S^-1^, with an R^2 ^value 0.99. Fluorescence quenching studies were carried out by using ethidium bromide (EB) bound herring sperm DNA with increasing concentrations of CuNPs [30]. The fluorescence quenching revealed a reasonable agreement with the classical stern-Volmer equation(1)

where I_FO _and I_F _are the emission intensity in the absence and presence of the quencher, respectively, Ksv is the stern - Volmer quenching constant and [CuNPs] is the concentration of CuNPs (Figure 3c). The value of Ksv was calculated as 0.00264 M^-1^. This result demonstrates that there is a significant interaction of CuNPs with the DNA. The apparent binding constant (K_app_) for DNA-CuNPs interaction was also calculated as 3.137 × 10^4 ^M^-1 ^using the following equation(2)

Where K_EB _= 1.0 × 10^7 ^M^-1^, [EB] = 1.3 μM and [CuNPs]50 is the concentration that cause a 50% quenching of the initial EB fluorescence [30].

**Figure 4:**
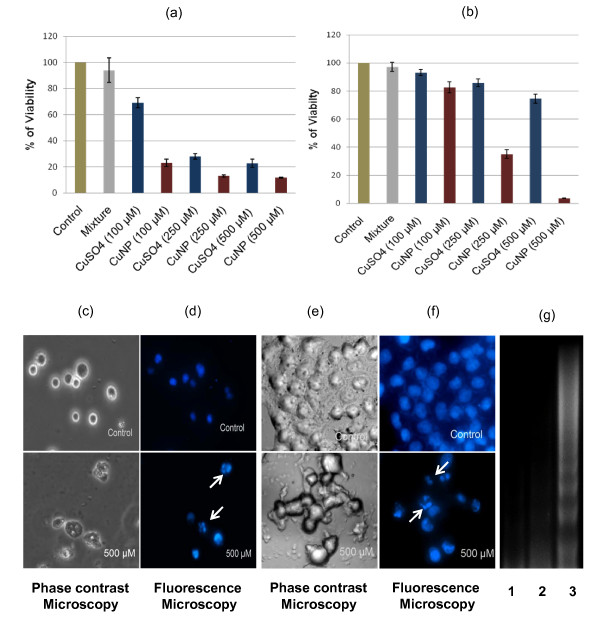
**Cytotoxic effect and induction of apoptosis by CuNPs towards U937 and Hela cells**. (a) U937 and (b) Hela cells were treated with 100, 250 and 500 μM of CuNPs, and CuSO_4 _for 24 hours. Cell viability based on MTT assay is shown where viability for control untreated cells was considered as 100%. Data are presented as Mean ± SE. Phase contrast microscopic pictures of (c) U937 and (e) Hela cells untreated (top) or treated with 500 μM CuNPs (bottom). Fluorescence microscopic pictures of DAPI stained (d) U937 and (f) Hela cells untreated (top) and treated with 500 μM CuNPs (bottom). Arrows indicate fragmented nuclei. (g) U937 cells were treated for 24 hours with 250 μM CuNPs (lane 3) or a mixture of sodium borohydride and sodium citrate (lane 2) and inter nucleosomal DNA fragmentation was analyzed by electrophoresis on a 1.6% Tris-Borate-EDTA agarose gel. Lane 1 represents untreated U937 cells.

Additionally, the effect of CuNPs on cultured U937 and Hela cells was tested. U937 cells were grown in RPMI-1640 medium and Hela cells were grown in DMEM medium in the presence of 10% fetal bovine serum under 5% CO_2 _in a humidified incubator at 37°C and were treated with different concentrations of CuNPs. U937 and Hela cells were also treated with a mixture of sodium borohydride and sodium citrate, and CuSO_4 _solutions as controls. Initially, to check the cytotoxic effect of CuNPs on U937 and Hela cells, a number of viable cells after exposure with CuNPs were enumerated by colorimetric MTT assay [31]. Percentages of surviving cells to untreated controls were calculated by using the formula as % viability = [(A_t_/A_s_) × 100] %, where A_t _and A_s _indicate the absorbance of the sample and control, respectively. Interference of copper in MTT assay was monitored and it was found that copper has an interfering effect with a maximum value of 17% increase in color production if it is considered that all of the added CuNPs (500 μM) enter inside the treated cells and are converted to Cu^+2 ^ions. Results of MTT assays (Figure 4a and 4b) clearly revealed the cytotoxic effect of CuNPs in a dose dependent manner for both the cell lines and CuNPs exerted slightly better cytotoxic effect towards Hela cells in comparison to U937 cells. Although, CuSO_4 _also showed cytotoxicity towards cancer cells, but the effect was much less compared to citrate protected CuNPs.

Cytotoxicity of metallic copper nanoparticles, copper oxide nanoparticles and ionic copper on different cells was documented earlier [12,32,33]. Studer et al. specifically compared cytotoxic effect of metallic copper nanoparticles, copper oxide nanoparticles and ionic copper on Chinese Hamster Ovary (CHO) cells and Hela cells [12]. It was observed that cytotoxic effect of carbon protected copper nanoparticles (C/Cu) towards CHO cells was less compared to CuO nanoparticles, but was greater than that of CuCl_2 _[12]. In contrast, Studer et al. found that in case of Hela cells, C/Cu could not exert significant cytotoxicity while both CuO nanoparticles and CuCl_2 _exerted cytotoxic effect [12]. Interestingly, in our present study, the citrate protected copper nanoparticles were able to show significant cytotoxicity towards both U937 and Hela cells as compared to CuSO_4_. In addition, U937 and Hela cells, after treatment with CuNPs, exhibited ultra structure and biochemical features that are characteristic of apoptosis, as shown by chromatin condensation and inter nucleosomal DNA fragmentation. The phase-contrast microscopic pictures of altered morphology of U937 and Hela cells which is characteristic of apoptotic cell stage when treated with CuNPs are shown in Figure 4c and 4e. Fluorescent microscopic studies after 4', 6-diamidino-2-phenylindole (DAPI) staining of untreated and CuNPs treated cells clearly exhibited nuclear fragmentation in CuNPs treated U937 and Hela cells which is a hallmark of cellular apoptosis (Figure 4d and 4f). Moreover, CuNPs treated U937 cells displayed a ladder pattern of inter nucleosomal DNA fragmentation on TBE-agarose gel electrophoresis in DNA ladder assay [34] as shown in Figure 4g (lane 3) which is also another hallmark of apoptosis. All these results demonstrate that treatment with CuNPs induce apoptosis in U937 and Hela cells.

**Figure 5:**
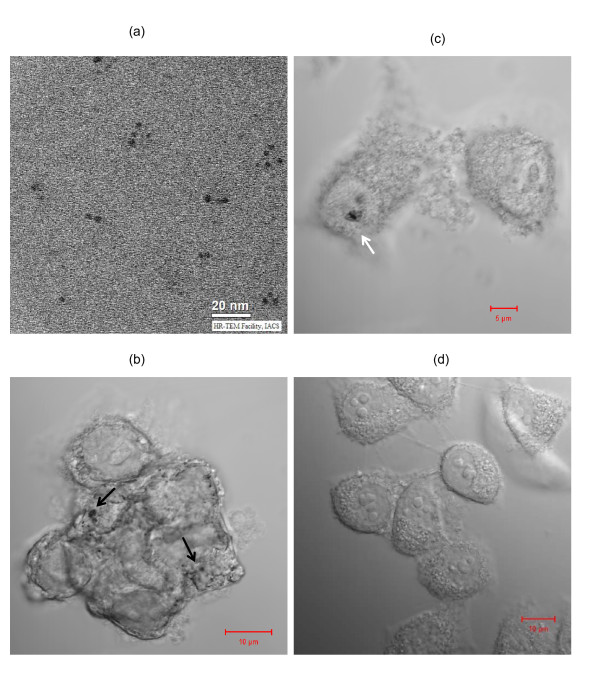
**Stability of Copper nanoparticles and uptake of citrate protected copper nanoparticles (CuNPs) in Hela cells**. (a) TEM image of copper nanoparticles incubated in DMEM medium for 3 hours. (b) Confocal microscopic image of Hela cells treated with 500 μM of Copper nanoparticles for 14 hours. Arrows indicate agglomerated CuNPs. (c) Confocal microscopic image of Hela cells treated with 500 μM of Copper nanoparticles for 14 hours. Arrow indicates agglomerated CuNPs in a membrane bound vesicle (probably perinuclear lysosome). (d) Confocal microscopic image of control Hela cells.

To check stability of copper nanoparticles we carried out a number of UV-Vis spectroscopy measurements and TEM studies. TEM study clearly revealed that the size of the nanoparticles remains similar after incubation of CuNPs in cell culture media indicating the stability of copper nanoparticles with respect to its agglomeration tendency in the cell culture medium (Figure 5a). However, the UV-Vis spectroscopy of CuNPs in cell culture media indicates slight agglomeration (Additional file 1 Figure S4). Confocal microscopic studies confirmed the uptake of CuNPs inside the Hela cells (Figure 5b and 5c) with the presence of agglomerated copper nanoparticles; a similar observation was also reported by Stark and coworkers with carbon coated copper nanoparticles for Hela cells [12].

In summary, it was observed for the first time that the copper nanoparticles can initiate the DNA degradation process and also can induce apoptotic cell death in cancer cells. The CuNPs degrade DNA in a singlet oxygen mediated fashion even in the absence of any external agents like hydrogen peroxide or ascorbate. This makes CuNPs as an excellent candidate for targeted therapy. The use of copper nanoparticles as therapeutic agents could be in particular advantageous because human body has an efficient system to deal with metabolism of copper since it is a micronutrient. So the residual copper expected to be produced during the nanoparticle based drug metabolism can be easily managed by the body. Furthermore, this DNA degradation potential and cytotoxic effect of CuNPs can be utilized in designing better and more active cancer drugs by chemically modifying the CuNPs with a number of macromolecules. Current efforts in our laboratory are underway to address these questions and to study the molecular mechanisms of CuNPs mediated cytotoxicity through apoptosis towards cancer cells of different origins.

## Competing interests

The authors declare that they have no competing interests.

## Authors' contributions

SS synthesized and characterized copper nanoparticles. GPJ performed experiments. SKM and TKS conceived and designed the experiments. GPJ, SS, SKM and TKS interpreted the data and prepared the manuscript. All authors read and approved the manuscript.

## Supplementary Material

Additional file 1 **Additional Data Files**. The file is organized into two sections. Section 1 describes essential methods. Section 2 provides graphical representation of change in the percentage of the super coiled DNA on incubation with copper nanoparticles fitted in to an exponential decay function (Figure S1) and Emission spectra of Ethidium Bromide bound to DNA in the absence and presence of different concentrations of copper nanoparticles (Figure S2). Figure S3 represents UV-Vis spectroscopic profile of CuNP (250 μM) incubated in PBS, pH 7.4 for different times at 37°C. Figure S4 represents UV-Vis spectroscopic profile of (A) CuNPs (250 μM) and (B) CuSO_4 _(250 μM) incubated in DMEM cell culture medium for different times at 37°C. Click here for file
