# Supplementary material for: Singlet oxygen mediated DNA degradation by copper nanoparticles: potential towards cytotoxic effect on cancer cells
Source: J Nanobiotechnology. 2011 Mar 25;9:9. doi: 10.1186/1477-3155-9-9 (PMC3072310; doi:10.1186/1477-3155-9-9)
Supplement: Additional file 1 — Additional Data Files. The file is organized into two sections. Section 1 describes essential methods. Section 2 provides graphical representation of change in the percentage of the super coiled DNA on incubation with copper nanoparticles fitted in to an exponential decay function (Figure S1) and Emission spectra of Ethidium Bromide bound to DNA in the absence and presence of different concentrations of copper nanoparticles (Figure S2). Figure S3 represents UV-Vis spectroscopic profile of CuNP (250 μM) incubated in PBS, pH 7.4 for different times at 37°C. Figure S4 represents UV-Vis spectroscopic profile of (A) CuNPs (250 μM) and (B) CuSO4 (250 μM) incubated in DMEM cell culture medium for different times at 37°C. [file 1477-3155-9-9-S1.DOC]

**Additional Data Files**

### Section 1: Methods

**1.1 Preparation and characterization of CuNPs**

CuNPs, stabilized with citrate were prepared following a method described by Samim *et al* [1] in a modified way. Characterization of CuNPs was carried out spectrophotometrically (HITACHI UV 4100 spectrophotometer) and also by transmission electron microscopy (TEM) (JEOL, JEM 2010/111).

**1.2. DNA concentration measurement**

The absorbance of genomic and plasmid DNA were measured at 260 and 280 nm using Beckman DU 730 spectrophotometer. The DNA concentration estimation was based on the extinction coefficient for absorbance at 260 nm (1 cm path length) of 0.02 µg mL-1 or DNA with an absorption 1 = 50µg mL-1.[2] The 260/280 ratio was near to 1.8 indicating good quality of the DNA. In the case of herring sperm DNA, 12858 M-1 taken as extinction coefficient at 260 nm and expressed the concentration in terms of base molarity. [3]

**1.3 *In vitro* degradation of bacterial genomic DNA**

Isolated DNA was mixed with varying concentrations (50-500 μM) of CuNPs in phosphate buffered saline, pH 7.4 and incubated at 37oC for 100 minutes. CuNPs solution was centrifuged at 260000 x g for 30 minutes at 4oC and the supernatant was used as a major control along with constituents of the CuNPs preparation separately. Soon after the incubation DNA samples were analyzed by agarose gel electrophoresis and visualized using Biorad gel documentation system.

For the detection of reactive oxygen species involved in CuNPs mediated DNA degradation, chemical scavengers Tris [hydroxyl methyl] amino methane, sodium azide, dimethyl sulphoxide, and D-mannitol were used separately in the degradation reaction mixtures.

**1.4 *In vitro* plasmid DNA degradation and rate constant measurement**

pET 28 b plasmid DNA was treated with 500 µM CuNPs for different time periods at 37 0C. 0.2 M of tris [hydroxyl methyl] amino methane was used to stop the reactions. Controls were taken as DNA alone, DNA with 0.2 M tris [hydroxyl methyl] amino methane, and 500 µM CuNPs. The separation and visualization of DNA bands were performed as mentioned above. The band intensity was measured using “quantity one” software. The super-coiled plasmid DNA due to its compact structure was corrected by multiplying the intensity with 1.42.The intensity of different forms of plasmid was plotted against the time in seconds.

**1.5 Fluorescence spectroscopic studies**

Interaction of CuNPs with isolated DNA molecules was studied by fluorescence spectroscopy (Hitachi F-7000) based on the competitive displacement of the intercalated Ethidium bromide from the DNA by increasing amounts CuNPs. Samples were excited at 530 nm and emission peak was monitored from 500-650 nm at room temperature. Maximum emission intensity was observed at 609 nm. Stern-Volmer plot was drawn by taking IFO/IF values on the Y axis and copper nanoparticle concentrations on the X axis. Stern-Volmer constant ( Ksv) was calculated form the slope of the plot. Kapp is calculated by taking 414.40 µM as [CuNPs]50.

**1.6 Cell culture maintenance**

U 937 cells and Hela cells were maintained in RPMI 1640 and DMEM media, respectively in tissue culture flasks, supplemented with 10% FBS, 100 units/ml penicillin and100µg/ml streptomycin, temperature 37oC, 5% CO2­­.

## 1.7 MTT assay

## This assay is based on the reduction of tetrazolium salt MTT [3-(4,5-dimethylthiazol-2-yl)-2,5-diphenyltetrazolium bromide] into purple colored formazan by mitochondrial enzymes present in the live cells. The extent of reduction is directly proportional to the number of live cells present in the sample. To set up the assay, U937 cells (2X104­­ cell/well) and Hela cells (5000 cells/ well, incubated for overnight to allow the cells to get attached with the plate) were seeded in to a 96 well plate (Nunc) and treated with varying concentrations of Copper nanoparticles for 24 hours. After the incubation 20 µl of MTT solution (5mg/ml in PBS, filtered with 0.2 µ filter) was added and incubated for 2.5 hours at 37oC, 5% CO2­­. After the incubation, the plates were centrifuged at 0.6 RCF, 40C for 10 minutes. The supernatant was removed and added 200 µl DMSO and the purple colour developed due to the solubilisation of formazan crystals was estimated at 515 nm with only DMSO as blank using ELISA reader (ELx800, BioTek).

**1.8 Nuclear morphological change study using 4’, 6-diamidino-2-phenylindole (DAPI)**

In this study nuclear staining dye DAPI (4’, 6-diamidino-2-phenylindole) was used to study the morphological changes in the nucleus of U 937 cells on the treatment of copper nanoparticles. U937 cells (0.5 million/ml) were seeded in to 6 well plate and varying concentrations of copper nanoparticles were added and incubated for 24 hours at 37oC, 5% CO2­­. After the incubation cells were centrifuged at 0.6 RCF for 5 minutes and removed the supernatant. Added 200µL of PBS buffer pH 7.4 and 0.8µL of DAPI from a stock of 1 mg/ml to make a final concentration 4 µg/ml. Samples were incubated under dark for 15 minutes. The nuclear stained cells were observed in bright field and epifluorescence microscopy.

**1.9 DNA ladder assay**

U 937 cells (0.5million/ml) were treated with different concentrations of copper nanoparticles for 24 hours. After the incubation cells were centrifuged at 0.6 RCF for 5 minutes and resuspended in 0.5 ml of resuspension buffer (20 mM Tris, 0.4mM EDTA, 0.25% Triton X 100) and incubated at room temperature for 15 minutes. It was followed by centrifugation at 14,000 rpm at 40C for 10 minutes. 55 µL of sodium chloride (5M) and 550 µL of isopropanol were added to the supernatant and incubated at -200C overnight. After the incubation, the supernatant was centrifuged at 14,000 rpm at 40C for 10 minutes and the pellet was washed with 70% alcohol followed by drying the pellet at room temperature. The dried pellet was dissolved in 20 µL of TE buffer and the inter nucleosomal DNA fragments were separated using 1.6% agarose gel in TBE buffer.

**1.10 Copper nanoparticle internalization study using confocal microscopy**

Hela cells were grown on glass cover slips in DMEM medium and treated with 500 µM CuNP for 14 hours. After the treatment, cells were fixed using 4% paraformaldehyde solution in PBS at 4oC for 2 hours. It was followed by washing with PBS and stained with DAPI (2µg/mL) to visualize the nucleus. These glass cover slips were further washed again with PBS and mounted on to glass slides. Confocal imaging of the cells were carried out using standard confocal laser scanning microscope (LSM 710; Zeiss) in T-PMT ( transmitted light PMT) mode using 100x oil immersion objective. Fluorescent imaging was carried out in the same microscope using 405 nm excitation.

**References:**

[1] Samim M, Kaushik NK, Maitra A: *Bull Mater Science* 2007, **30:** 535–540.

[2] M. J. Holden, S. A. Rabb, Y. B. Tewari, M. R. Winchester, *Anal. Chem.* **2007**, 79, 1536-1541.

[3] W. Zhong, J. Yu, Y. Liang, *Spectrochim. Acta, Part A* **2003**, *59*, 1281-1288.

## Section 2: Figures

**Figure S1.** Graph showing the change in the percentage of the super coiled DNA on incubation with copper nanoparticles fitted in to an exponential decay function.

**Figure S2**. Emission spectra of EB bound to DNA in the absece and presence of 20, 40, 60, 80, 100, 120, 140, 180, 220, 260, 300, 340, 380, 420, 460 and 500 µM of copper nanoparticles. The arrow shows the change in the intensity on increasing concentration of the nanoparticles.

(a)

(b)

**Figure S3.** UV-Vis spectroscopic profile of CuNPs (250 μM) recorded in PBS buffer at different time intervals indicating no appreciable change over the course of measurement (the black, red, blue and cyan color indicate the spectra recorded at 0 hour,1 hour, 2 hours and 3 hours, respectively).

**Figure S4.** UV-Vis spectroscopic profile recorded in DMEM medium at different time intervals (the red, blue and cyan color indicate the spectra recorded at 1 hour, 2 hours and 3 hours, respectively): (a) CuNPs (250 μM), the peak around 560 nm, characteristic of surface plasmon resonance peak due to presence of copper nanoparticles over 55 nm size indicates that copper nanoparticles undergo agglomeration and (b) CuSO4 (250 μM).
